# Patient satisfaction and wait times following outpatient manual vacuum aspiration compared to electric vacuum aspiration in the operating room: a cross-sectional study

**DOI:** 10.1186/s40834-017-0045-6

**Published:** 2017-06-07

**Authors:** Laura E. Dodge, Lisa G. Hofler, Michele R. Hacker, Sadia Haider

**Affiliations:** 1 Department of Obstetrics and Gynecology, Beth Israel Deaconess Medical Center, 330 Brookline Avenue, KS 3, Boston, MA 02215 USA; 2 Department of Obstetrics, Gynecology and Reproductive Biology, Harvard Medical School, 25 Shattuck St, Boston, MA 02115 USA

**Keywords:** Manual vacuum aspiration, Electric vacuum aspiration, Uterine evacuation, Patient acceptability

## Abstract

**Background:**

Outpatient manual vacuum aspiration (MVA) is a safe and equally effective alternative to electric vacuum aspiration (EVA) in the operating room. This project was conducted to determine whether outpatient MVA expedites care while maintaining patient satisfaction.

**Methods:**

A cross-sectional study of a convenience sample of patients undergoing surgical management of spontaneous abortion, induced abortion, or retained products of conception with either outpatient MVA under local anesthesia or EVA in the operating room was conducted. Of 138 women completing surveys, 48 (34.8%) underwent outpatient MVA and 90 (65.2%) underwent EVA in the operating room. Procedure length, time from decision to procedure, and patient satisfaction were assessed through a self-administered questionnaire completed post-procedure.

**Results:**

Most (77%) patients in the MVA group reported waiting fewer than 2 h from the time of their decision to the procedure, while most (74%) EVA patients reported waiting over 12 h (*P* < 0.001); the MVA group reported higher satisfaction with time to procedure (*P* = 0.02). The median procedure length was significantly shorter in the EVA group (10 vs. 20 min, *P* < 0.001). There was no significant difference between groups in overall satisfaction with the procedure (*P* = 0.16).

**Conclusion:**

Outpatient MVA under local anesthesia is a suitable alternative to operating room-based EVA for management of spontaneous abortion, induced abortion, and retained products of conception. Outpatient MVA is associated with shorter decision-to-procedure time and is highly acceptable to patients. Integration of outpatient MVA into clinical settings can add time- and resource-saving options for uterine evacuation while maintaining a positive patient experience.

## Background

Among the 1.1 million abortions performed each year, 77% are surgical procedures [8]. Electric vacuum aspiration (EVA) was the original surgical technique described in North America for performing surgical abortions [9, 10, 16]. Interest in manual vacuum aspiration (MVA) has grown, and its safety and efficacy for abortion care in the first trimester are well established [6, 17]. Use of MVA in an outpatient setting has distinct advantages over EVA in terms of cost [2]. Thus, MVA is now selectively used by half of outpatient abortion providers in the U.S. to provide access to abortion care for many women who otherwise could not afford it [11]. Additionally, previous work has demonstrated the acceptability of MVA among providers [3] and patients [1, 3, 4, 17], with one study showing that many women prefer the increased feeling of privacy afforded by treatment in the office setting [2].

While MVA can be used for any indication for uterine evacuation, its growing use may broaden access to induced abortion services, an increasingly critical issue in the current climate of expanding abortion restrictions [5] and a decreasing number of providers [8]. After a large hospital in Chicago moved its abortion services to an outpatient setting, the mean wait time for services fell from 20 to 10 days, and the mean gestational age decreased from 11.0 to 10.4 weeks [12]. Among this group, the introduction of MVA was associated with a 15% increase in the number of procedures per session [12].

Given these advantages, the aim of this project was to determine whether the introduction of outpatient MVA at an academic medical centre could expedite patient care compared to the typical practice of EVA in the operating room for the same indications while also maintaining patient satisfaction.

## Methods

In 2009, the Department of Obstetrics and Gynecology at our institution introduced outpatient MVA under local anaesthesia as an alternative to EVA with intravenous anaesthesia performed in the operating room for the treatment of spontaneous abortion, induced abortion, incomplete abortion, and retained products of conception. To assess the impact of this policy change, a convenience sample of women who chose surgical management of spontaneous abortion, induced abortion, incomplete abortion, and retained products of conception were surveyed after their procedure. Not all outpatient offices offered MVA. Women underwent MVA or EVA based on the location of their diagnosis. Women seen in the inpatient triage area and in outpatient offices that had MVA available underwent MVA procedures. Women diagnosed in outpatient offices without MVA available had EVA in the operating room. Our institution does not consider scheduled uterine evacuations “urgent” or “emergent” procedures; therefore, patients must fast prior to anaesthesia, and procedure timing often depends on operating theatre availability. Due to fasting requirements and operating room theatre availability, patients undergoing EVA generally go home to wait and then return for their procedure.

Women who underwent a scheduled uterine evacuation with a uterine size less than 12 weeks of gestation, were hemodynamically stable, were at least 18 years of age, and could proficiently read and write in English were eligible to participate. Prior to their procedure, eligible women were asked if they would complete a short self-administered questionnaire following their procedure. This questionnaire assessed demographic characteristics, the time from when they made their decision for uterine evacuation to the time they underwent the procedure, and their satisfaction with the procedure. Statements regarding specific aspects of the procedure (e.g., “the number of staff involved in my care was appropriate,” “I had the procedure quickly after the diagnosis or decision”) were scored on a five-point Likert scale, with ‘1’ being ‘strongly agree’ and ‘5’ being ‘strongly disagree.’

Procedure characteristics such as procedure length, estimated blood loss, and concurrent intrauterine contraceptive device placement were obtained from the medical record. Procedure length was defined as the time from speculum insertion to speculum removal. The Committee on Clinical Investigations at Beth Israel Deaconess Medical Center determined this was a quality assurance project and thus exempt from review.

Data are presented as medians with interquartile range and counts with proportion. Comparisons of continuous variables were made using the Wilcoxon rank sum test, and comparisons of categorical variables were made using Fisher’s exact test. A P value <0.05 was considered statistically significant. All data were analyzed using SAS 9.3 (SAS Institute, Cary, NC) and all tests were two sided.

## Results

One hundred thirty-eight women completed post-procedure surveys. Of these 138 women, 48 (34.8%) underwent outpatient MVA under local anaesthesia, and 90 (65.2%) underwent EVA in the operating room with either intravenous sedation or general anaesthesia (Table 1). There were no differences between women who underwent MVA and those who underwent EVA with respect to age, education, race/ethnicity, gravidity, or parity (all *P* ≥ 0.29). However, women did differ with regards to the specialty of the treating attending physician; women undergoing MVA were more likely to be treated by a family planning specialist than a general obstetrician-gynaecologist (*P* < 0.001).

**Table 1:** Patient characteristics

Characteristic	Electric vacuum aspiration *n* = 90	Manual vacuum aspiration *n* = 48	*P*
Age (years)	33.4 (26.9–37.2)	32.3 (27.7–36.1)	0.98
Education			0.87
*High school or less*	11 (12.9)	7 (14.6)	
*Some college*	17 (20.0)	11 (22.9)	
*College degree or more*	57 (67.1)	30 (62.5)	
Race/ethnicity			0.29
*Caucasian*	44 (51.2)	24 (52.2)	
*African American*	17 (19.8)	15 (32.6)	
*Asian*	8 (9.3)	1 (2.2)	
*Hispanic/Latina*	9 (10.5)	4 (8.7)	
*Other*	8 (9.3)	2 (4.4)	
Gravidity			0.45
*1*	28 (31.3)	12 (25.0)	
*2 or more*	62 (68.9)	36 (75.0)	
Parity			0.81
*0*	39 (43.8)	20 (41.7)	
*1 or more*	50 (56.2)	28 (58.3)	
Vaginal parity			0.20
*0*	15 (32.6)	9 (32.1)	
*1*	21 (45.7)	8 (28.6)	
*2 or more*	10 (21.7)	11 (39.3)	
Attending of record			<0.001
*Family planning*	35 (38.9)	35 (72.9)	
*General Ob-Gyn*	55 (61.1)	13 (27.1)	

Data are shown as median (interquartile range) or n (%)

*Ob-Gyn* obstetrician-gynaecologist

Procedure characteristics differed significantly between women undergoing MVA and those undergoing EVA with regards to indication for uterine evacuation (Table 2). Missed abortion was a more common indication in the EVA group (52.2% vs. 14.6%), and induced abortion was a more common indication in the MVA group (58.3% vs. 38.9%; *P* < 0.001).

**Table 2:** Procedure characteristics

Characteristic	Electric vacuum aspiration (EVA) *n* = 90	Manual vacuum aspiration (MVA) *n* = 48	*P*
EBL	10.0 (0.0–50.0)	20.0 (20.0–25.0)	0.05
Length of the procedure	10.0 (8.0–13.0)	20.0 (15.0–25.0)	<0.001
Time in the OR (EVA) or office (MVA)	32.5 (27.0–37.0)	130 (115–165)	<0.001
Concomitant IUD insertion	0 (0.0)	7 (33.3)	0.002
Sharp curettage used	24 (26.7)	1 (2.1)	<0.001
Indication			<0.001
*Missed abortion*	47 (52.2)	7 (14.6)	
*Induced abortion*	35 (38.9)	28 (58.3)	
*rPOC after spontaneous abortion*	4 (4.4)	9 (18.8)	
*rPOC after induced abortion*	1 (1.1)	3 (6.3)	
*Other*	3 (3.3)	1 (2.1)	
Patient wait times			<0.001
*< 2 h*	13 (14.9)	36 (76.6)	
*2*–*6 h*	7 (8.1)	3 (6.4)	
*6*–*12 h*	3 (3.5)	1 (2.1)	
*> 12 h*	64 (73.6)	7 (14.9)	
Patient left hospital/office after decision was made and returned for the procedure	84 (94.4)	13 (27.7)	<0.001

Data are shown as median (interquartile range) or n (%)

*IUD* intrauterine device, *rPOC* retained products of conception

Most (76.6%) women in the MVA group reported waiting fewer than 2 h from the time of their treatment decision to the time of the procedure, while most (73.6%) EVA patients reported waiting over 12 h (*P* < 0.001). Nearly all (94.4%) women undergoing EVA left the hospital or clinic after making the decision to have surgery and returned later for their procedures, whereas only 27.7% of women undergoing MVA left the hospital or clinic prior to having their procedures (*P* < 0.001). Total procedure time was 10.0 (8.0–13.0) minutes among women undergoing EVA and 20.0 (15.0–25.0) minutes among women undergoing MVA (*P* < 0.001); there was no difference in procedure time between family planning specialists and general obstetrician-gynaecologists overall (*P* = 0.27) or in the MVA (*P* = 0.78) or EVA (*P* = 0.16) group. Notably, one third of women undergoing MVA had intrauterine contraceptive devices placed during the procedure, which was included in the overall procedure time, while no patients undergoing EVA underwent concurrent intrauterine device insertion.

Overall, there was no difference in the proportion of women who reported being very satisfied with the procedure in the EVA group (77.8%) and the MVA group (62.5%; *P* = 0.06; Table 3). EVA patients did not have the option to have a support person present during their procedure, but nearly all (95.7%) women undergoing MVA strongly agreed or agreed that they liked having this option available. While there was no difference in patients’ preference for location (hospital-based operating room or outpatient office; *P* = 0.35), women undergoing MVA were significantly more likely than those undergoing EVA to agree that the time to their procedure was reasonable (*P* = 0.02).

**Table 3:** Patient satisfaction

Characteristic	Electric vacuum aspiration *n* = 90	Manual vacuum aspiration *n* = 48	*P*
Agreed with the statements^a^			
*Staff number was appropriate*	1.0 (1.0–1.0)	1.0 (1.0–1.0)	0.51
*Time to procedure was reasonable*	2.0 (1.0–2.0)	1.0 (1.0–2.0)	0.02
*Liked the location*	1.0 (1.0–2.0)	1.0 (1.0–2.0)	0.35
*Liked having a support person*	--	1.0 (1.0–1.0)	--
Perception of the procedure			<0.001
*Better than expected*	63 (72.4)	16 (33.3)	
*Worse than expected*	0 (0.0)	9 (19.8)	
*Same as expected*	24 (27.6)	23 (47.9)	
Satisfaction			
*Very satisfied*	70 (77.8)	30 (62.5)	0.06
*Satisfied*	16 (17.8)	14 (29.2)	0.12
*Neutral*	4 (4.4)	4 (8.3)	0.45

Data are shown as median (interquartile range) or n (%)

^a^Statements were scored on the following five-point Likert scale: 1, strongly agree; 2, agree; 3, neutral; 4, disagree; and 5, strongly disagree

## Discussion

This study demonstrates that outpatient MVA is associated with significantly shorter patient wait times and increased patient satisfaction with their wait times. There was no difference between the groups in the proportion of women who reported being highly satisfied with their procedure. Although we found significantly shorter procedure durations for women undergoing EVA compared to MVA, prior research has been mixed. Several studies have found no significant differences between MVA and EVA procedure times in first and second trimester procedures [3], but a systematic review showed that EVA may have shorter procedure times due to the need to empty the MVA syringe during the procedure [17]. The differences in procedure times in our study may also be due to concomitant intrauterine device placement for one third of MVA patients. EVA procedure times did not include time in the preoperative area, receiving anaesthesia, and recovering from anaesthesia, all of which take longer in the peri-operative setting than in the outpatient office.

As access to induced abortion becomes increasingly restricted throughout the country, it is important to maximize access where possible. One way of maximizing access is by increasing the number of procedures that can be performed in a single centre, as was shown with a 15% increase in capacity when a large Chicago hospital moved their abortion procedures to an outpatient facility [12]. Additionally, integration of MVA into the outpatient setting can allow other providers, e.g., mid-level providers and family medicine physicians, to provide increased access to care for women seeking induced abortion and also treat women who require surgical management of spontaneous or incomplete abortion.

The use of MVA can also improve the patient experience by allowing shorter wait times prior to the procedure. Women who choose surgical management of miscarriage or retained products of conception likely want to have the procedure as soon as possible. By shortening the wait time, the gestational age at the time of induced abortion also decreases as women can be treated sooner, and larger decreases in wait time may be associated with fewer complications [13] and less patient discomfort [14]. This may also impact women in states with gestational age restrictions on abortion, as even a 1-day delay can be the difference between being able to access a procedure and pay for it versus the inability to safely access abortion care. This is especially important for women whose care is delayed until the second trimester, as terminations in the second trimester are associated with increased cost and complications and decreased access, as many states require all second-trimester abortions to be performed in a hospital or ambulatory surgical centre [7, 13]. Given the 10-day difference in wait times shown in the study by Patel et al., increasing access to terminations in the outpatient setting by multiple provider types could markedly improve timely access to abortion care [12].

Finally, MVA has the potential to save costs for the institution. A cost-effectiveness model examining different care strategies for uterine evacuation in early pregnancies estimated that in the U.S., MVA could save $779 million per year compared to EVA [15]. Moving uterine evacuation to the office saved nearly $1,000 per case in the study by Dalton et al., while physician reimbursement did not differ [2].

## Conclusion

This study demonstrates that outpatient MVA under local anaesthesia is a suitable alternative to EVA in the operating room for management of spontaneous and induced abortion, as well as retained products of conception. Outpatient MVA is associated with shorter time from diagnosis to procedure and is acceptable to patients. Integration of outpatient MVA into clinical settings has the ability to save resources while maintaining a positive patient experience and expediting patient care. Further integration of outpatient MVA may be an important strategy to maintain access to induced abortion services in the setting of increased abortion restrictions. Additionally, this would allow women undergoing management of spontaneous abortion or incomplete abortion to access care in a timely fashion within the comfort of their provider’s office.

## Abbreviations

EVA: Electric vacuum aspiration
MVA: Manual vacuum aspiration
